# The Broad Autism (Endo)Phenotype: Neurostructural and Neurofunctional Correlates in Parents of Individuals with Autism Spectrum Disorders

**DOI:** 10.3389/fnins.2016.00346

**Published:** 2016-07-22

**Authors:** Lucia Billeci, Sara Calderoni, Eugenia Conti, Camilla Gesi, Claudia Carmassi, Liliana Dell'Osso, Giovanni Cioni, Filippo Muratori, Andrea Guzzetta

**Affiliations:** ^1^Department of Clinical and Experimental Medicine, University of PisaPisa, Italy; ^2^IRCCS Stella Maris FoundationPisa, Italy; ^3^Department of Sciences for Health Promotion and Mother and Child Care G. D'Alessandro, University of PalermoPalermo, Italy

**Keywords:** Autism Spectrum Disorders, parents, Broader Autism Phenotype, magnetic resonance imaging, magnetic resonance spectroscopy, electroencephalography, magnetoenchephalography

## Abstract

Autism Spectrum Disorders (ASD) are a set of neurodevelopmental disorders with an early-onset and a strong genetic component in their pathogenesis. According to genetic and epidemiological data, ASD relatives present personality traits similar to, but not as severe as the defining features of ASD, which have been indicated as the “Broader Autism Phenotype” (BAP). BAP features seem to be more prevalent in first-degree relatives of individuals with ASD than in the general population. Characterizing brain profiles of relatives of autistic probands may help to understand ASD endophenotype. The aim of this review was to provide an up-to-date overview of research findings on the neurostructural and neurofunctional substrates in parents of individuals with ASD (pASD). The primary hypothesis was that, like for the behavioral profile, the pASD express an intermediate neurobiological pattern between ASD individuals and healthy controls. The 13 reviewed studies evaluated structural magnetic resonance imaging (MRI) brain volumes, chemical signals using magnetic resonance spectroscopy (MRS), task-related functional activation by functional magnetic resonance imaging (fMRI), electroencephalography (EEG), or magnetoencephalography (MEG) in pASD.The studies showed that pASD are generally different from healthy controls at a structural and functional level despite often not behaviorally impaired. More atypicalities in neural patterns of pASD seem to be associated with higher scores at BAP assessment. Some of the observed atypicalities are the same of the ASD probands. In addition, the pattern of neural correlates in pASD resembles that of adult individuals with ASD, or it is specific, possibly due to a compensatory mechanism. Future studies should ideally include a group of pASD and HC with their ASD and non-ASD probands respectively. They should subgrouping the pASD according to the BAP scores, considering gender as a possible confounding factor, and correlating these scores to underlying brain structure and function. These types of studies may help to understand the genetic mechanisms involved in the various clinical dimension of ASD.

## Introduction

Autism Spectrum Disorders (ASD) are a set of early-onset neurodevelopmental disorders that are characterized by a disrupted development of brain connectivity with several cascading effects on neuropsychological functions (Narzisi et al., 2013; Kana et al., 2014). A clinical dyad, comprising social communication difficulties and repetitive, stereotyped behavior must be present for a diagnosis of ASD (American Psychiatric Association, 2013). The exact cause of ASD is still unknown (Levy et al., 2009). Although, only 20% of ASD cases can be explained by a specific genetic cause, such as identifiable genetic syndromes, genetic mutations or de novo copy number variants (Jeste and Geschwind, 2014), recent twin studies estimate an heritability between 64 and 91% (Tick et al., 2016), suggesting an interaction between genetic vulnerability and environmental factors (Rossignol et al., 2014).

Genetic epidemiological data suggest that personality traits similar to, but not as severe as those of ASD, are also heritable (Freitag, 2007). This group of “sub-threshold” features, which are believed to be milder manifestations of ASD (Dell'Osso et al., 2016), have been indicated as the broader autism phenotype (BAP) (Piven et al., 1997). BAP includes peculiar social, communication, and cognitive processes, strong persistent interests, and rigid and aloof personality traits (Gerdts and Bernier, 2011; Sucksmith et al., 2011). Interestingly, it was shown that BAP traits are more prevalent in first-degree relatives of individuals with ASD than in other groups, supporting the hypothesis that ASD have a significant genetic component (Bailey et al., 1998; Losh et al., 2008).

Kanner and Asperger were the first to report behavioral features in parents that were similar in kind to those of their autistic offspring. In particular, Kanner (1943) observed that both first and second degree relatives of children with “early infantile autism” had common characteristics of late speech, mild obsessiveness and uninterest in people. Similarly, Asperger (1944) described a group of parents of children with autism as withdrawn, pedantic, eccentric, and loners, who had problems relating to the outside world. Later studies have shown that the expression of ASD traits in relatives concerns not only behavioral traits, but also social cognition abilities (e.g., Baron-Cohen and Hammer, 1997), neurocognitive functioning (e.g., Koczat et al., 2002) or biological dimensions (e.g., Lainhart et al., 2006) and that these aspects could relate to or explain the clinical presentation of the BAP.

The biological dimension of ASD has been largely investigated in the last decades, thanks to the growing availability of brain imaging techniques and analysis methods for *in vivo* examination of brain structure and function. All in all, these studies reported abnormal neuroanatomical and neurofunctional profiles in individuals with ASD, suggesting a dysfunction of key brain areas underlying the core impairments of ASD (Amaral et al., 2008; Bellani et al., 2013a,b; Billeci et al., 2013; Calderoni et al., 2014). As such, there has been great interest in evaluating whether these neurological profiles also characterize the relatives of autistic probands. Indeed, should the same brain abnormalities of ASD patients be present in their direct relatives, their heritable origin would be strongly supported together with their role as endophenotypes of the disorder (Sullivan et al., 2003; Palmen et al., 2005a). This is particularly true for studies exploring correlations in parents. In fact, while sibling and twin studies are suitable for detecting brain abnormalities under genetic control, studies on parents allow mitigating the role of the shared (pre- and perinatal) environment (Sullivan et al., 2003; Palmen et al., 2005a). Thus, if brain abnormalities are observed in parents, they are more likely to be of heritable origin and consequently reflect endophenotypes of the disorder. To assess the strength of this hypothesis, we provide here a critical revision of all studies exploring the neuroanatomical and neurofunctional profile of parents of individuals with ASD.

## Methods

To find papers concerning neuroimaging studies in parents of individuals with ASD, a sensitive search strategy was conducted in two relevant article databases: PubMed and ScienceDirect. Search terms included database subject headings for the concepts of pervasive developmental disorders (e.g. “autism,” “autism spectrum disorder,” “pervasive developmental disorders”), neuroimaging (e.g., “MRI,” “MRS,” “EEG,” “MEG”) and parents (“parents,” “relatives,” “fathers,” “mothers,” “broader phenotype”). The reference lists of the retrieved papers were searched to identify additional articles.

Studies adhering to the following criteria were incorporated in this review: (1) parents of individuals with ASD were the population under study; (2) Magnetic Resonance Imaging (MRI), Magnetic Resonance Spectroscopy (MRS), Electroencephalography (EEG) and Magnetoencephalography (MEG) were used to investigate neurostructural and neurofunctional correlates in parents of individuals with ASD; (3) empirical findings about neural substrates were reported by the authors; (4) studies were published before March 30, 2016; (5) studies were published in an English peer-reviewed journal.

## Results

Thirteen published studies meeting the inclusion criteria were identified. Table 1 summarizes the studies included in this review.

**Table 1:** **Neuroanatomical and neurofunctional characteristics associated to the BAP in the parents of individuals with ASD**.

**Study (year)**	**Participants (nr, M/F, mean age ± SD in years)**	**BAP Questionnaires**	**Method**	**Results**	**Correlations with BAP scores and behavior**
**STRUCTURAL STUDIES**					
Rojas et al., 2004	15 ASD (6/9) 30.3 ± 9.1	None	ROI manual tracing (HYP, AMY total brain)	ASD>pASD>HC left HYP	–
	17 pASD (15/2) 44.75 ± 4.4				
	17 HC (8/9) 43.6 ± 4.3			ASD<pASD, HC right AMY	
Palmen et al., 2005a	38 pASD (19/19) 50.3 ± 3.4	AQ	ROI semi-automatic tracing	No significant differences in volume	Positive correlations between AQ scores and intracranial and ventricular volume in pASD
	40 HC (20/20) 52.0 ± 4.1				
Peterson et al., 2006	23 pASD (8/15) 39.6 ± 6.0	None	VBM	pASD>HC in several GM regions (i.e. right precentral gyrus, right superior parietal lobule, and superior temporal gyri)	–
	23 HC (8/15) 38.3 ± 6.4			pASC<HC anterior portion of the left cerebellar hemisphere	
**fMRI STUDIES**					
Baron-Cohen et al., 2006	12 pASD (6/6) M: 39.1 ± 6.0	None	Visual Search Task (EFT) and Emotion Recognition Task (ET)	Females>Males>Fathers = Mothers in BA 19 in EFT task Females>Males>Fathers = Mothers in BA 21 e BA 44 in ET task	–
	F: 37.3 ± 5.9				
	12 HC (6/6) M: 23.1 ± 0.6				
	F: 21.6 ± 0.8				
Greimel et al., 2010	15 ASD (15/0) 14.9 ± 1.6	AQ	Empathy: other-task and self-task	pASD<pHC AMY other-task	No significant correlations between brain activity and AQ scores
				pASD<pHC FG other-task	
				ASD<HC FG other-task and self-task	
					Positive correlation between FG activation and GEM score in ASD
	15 HC (15/0) 15.0 ± 1.4			ASD<HC IFG self-task	
	11 pASD (11/0) 43.9 ± 5.1				
					Positive correlation between insula activation and BEES score in pASD and pHC
	9 pHC (9/0) 47.7 ± 5.3				
Wilson et al., 2013	16 pASD (6/10) 43.7 ± 8.1	AQ	Phonological processing: homophones vs pseudohomophone	pASD>HC pseudohomophone several regions (i.e. IC, STG, SMG, SMA, cerebellum)	Positive correlations between IFG activation and CTOPP scores in pASD and HC
	18 HC (6/12) 41.0 ± 8.1			pASD<HC left STG and left SMG phonological priming	
					Positive correlations between IC activation and CTOPP scores in pASD
Yucel et al., 2014	40 pASD (20/20) 40.6 ± 0.7	BAPQ	Face processing	pASD>HC AMY	BAP+>BAP−,HC LOC
		MPAS-R			
				pASD>HC FG	
	15 BAP+ 40.9 ± 1.4			pASD<HC INS	
	25 BAP− 42.1 ± 1.28				
	20 HC (6/12) 39.8 ± 1.6				
**MRS STUDIES**					
Brown et al., 2013	13 ASD (9/4) 41.2 ± 6.9	AQ	Level of Glu, NAA, Cr in auditory cortex	ASD>HC Glu, NAA, Cr	Positive correlation, uncorrected for multiple comparisons, between left NAA and the SRS and left Glu and the AQ
		SRS		No differences between pASD and ASD or HC	
	15 pASD (11/4) 41.0 ± 8.1				
	15 HC (6/9) 41.1 ± 6.8				
**EEG AND MEG STUDIES**					
Dawson et al., 2005	21 pASD (10/11) 38.5 ± n.d.	None	Face processing ERPs	pASD<HC N170 right amplitude to faces	Positive correlation between N170 amplitude to faces and WMS Immediate and Delay task in HC
				pASD<HC N170 latency difference chairs-faces	
	21 HC (8/13) 38.9 ± n.d.				
Rojas et al., 2008	11 ASD (9/2) 42.6 ± 5.1	None	Auditory stimulation	pASD,ASD>HC induced tGBR	–
			Evoked, induced and total power tGBR	pASD,ASD<HC evoked tGBR, PLF, anterior-posterior asymmetry	
	16 pASD (9/7) 31.5 ± 9.3				
			PLF tGBR	No differences between pASD and ASD	
			Source Localization		
	16 HC (7/9) 43.1 ± 6.7				
Rojas et al., 2011	21 pASD (7/13) 43.7 ± 7.3	AQ	Auditory stimulation	pASD<HC total and evoked power, PLF ASSR	Negative correlation between ASSR PLF and AQ communication subscale
	20 HC (6/15) 43.8 ± 6.9	SRS	Evoked, induced and total power tGBR	No differences in tGBR	
			PLF tGBR		Negative correlation between tGBR and ASSR evoked power and SRS scores
			Evoked, induced and total power ASSR		
			PLF ASSR		
McFadden et al., 2012	23 pASD (8/15) 35.8 ± 10.0	None	Language auditory stimulation	pASD>HC evoked and total gamma SMG, LOC	Significant but different correlations between gamma or beta activity and language measures (expressive, receptive, figurative language and phonological processing) in pASD and HC
			Evoked, induced and total power gamma and beta	pASD>HC evoked and total gamma SMG, LOC	
	28 HC (12/16) 38.7 ± 6.3				
			PLF gamma and beta	pASD>HC left lateralization	
Buard et al., 2013	12 ASD (?/?) 28.3 ± 13.3	None	Picture-naming task	ASD<HC high-gamma in right STG, evoked high-beta/low-gamma in left IFG and PLF beta in OCC	No significant correlation between MEG measures and language scores
	14 pASD (?/?) 37.9 ± 5.9				
			Evoked, induced and total power gamma and beta	pASD>HC high-gamma in left STG and evoked high-beta/low-gamma in left FG	
	35 HC (?/?) 34.2 ± 11.9				
			PLF gamma and beta	ASD>HC connectivity between IFG and FG and between STG and OCC in both gamma and beta band	
			Granger Causality		

*BAP, Broader Autism Phenotype; ASD, Autism Spectrum Disorders; HC, healthy controls; pASD, parents of individuals with ASD; SD, standard deviation*.

*AQ, Autism Quotient; BEES, Balanced Emotional Empathy Scale; BAPQ, Broad Autism Phenotype Questionnaire; MPAS-R, Modified Personality Assessment Schedule —Revised; SRS, Social Responsiveness Scale*.

*ROI, region of interest; VBM, voxel-based morphometry; EFT, “Adult Embedded Figures” test; ET, “Reading the Mind in the Eyes” (or Eyes) test; Glu, glutamate; NAA, n-acetyl aspartate + n-acetyl aspartyl; Cr, phosphocreatine and creatine; ERPs, evoked response potentials; tGBR, transient gamma-band response; ASSR, auditory steady-state response; PLF, phase locking factor; CTOOP, Comprehensive Test of Phonological Processing; WMS, Wechsler Memory Scale*.

*HYP, hippocampus; AMY, amygdala; GM, gray matter; BA, Broadmann area; FG, fusiform gyrus; IFG, inferior frontal gyrus; IC, insular cortex, STG, superior temporal gyrus, SMG, supramarginal gyrus; SMA, supplementary motor area; INS, insula; LOC, lateral occipital cortex; OCC, occipital lobe*.

### Structural MRI

Only three studies used sMRI to assess brain structure in parents of autistic probands.

Rojas et al. (2004) assessed total brain, hippocampus, and amygdala volumes in adults with ASD, parents of children with ASD (pASD) and healthy controls (HC), defined as adults with no personal or familial history of ASD. The left hyppocampus was found significantly larger in the ASD group in comparison to both the pASD and the HC group, and in the pASD group in comparison to the HC group. In the three groups, hyppocampus enlargement was more pronounced in males than in females. The right amygdala was smaller in the ASD group in comparison to both the pASD and the HC group, while no significant differences were found between pASD and HC. No differences were detected in the total brain volume among the three groups.

Palmen et al. (2005a) compared couples of pASD with known increased brain volumes with HC couples for volume differences in total brain, cortical lobes, cerebral and cortical gray matter (GM) and white matter (WM), cerebellum, and ventricles. The overt aim of the study was to investigate whether the cerebral enlargement observed in ASD probands (Palmen et al., 2005b) extended also to parents, and in this case whether fathers and mothers were equally affected and if the same regions, as those of the autistic probands, were interested in the enlargement. The authors found no group or gender differences in any of the brain volumes, including the volume of intracranium, total brain, GM and WM of the cerebrum, frontal, temporal, parietal, and occipital GM and WM, cerebellum, third and lateral ventricle. Nevertheless, within the pASD group significant positive correlations were found between the Autism Quotient (AQ) (Baron-Cohen et al., 2001a) scores and intracranial and ventricular volumes, suggesting that autistic traits might be associated to an enlargement in these structures.

In the third study, Peterson et al. (2006) compared regional GM volume in pASD and in HC, reporting an increase in several GM regions in pASD (e.g., superior temporal gyri, inferior and middle frontal gyri, superior parietal lobule, anterior cingulate). A single large relative decrease was observed in the anterior portion of the left cerebellar hemisphere in pASD compared with HC. Males showed increased GM compared with females in both groups, while no between-group differences respect to gender emerged.

It is worth noting that in the three above mentioned studies three different procedures were applied for data analysis. Specifically, Rojas et al. (2004) used manual tracing for selecting hippocampus and amygdala, Palmen et al. (2005a) applied a semi-automatic procedure to obtain a segmentation of the brain in the structure of interest and Peterson et al. (2006) applied an approach based on voxel-based morphometry (VBM).

### Magnetic resonance spectroscopy

Only one study used Magnetic Resonance Spectroscopy (MRS) to assess brain chemistry in parents of individuals with ASD (Brown et al., 2013). The aim of the study was therefore to determine whether the parents of ASD patients show higher levels of Glutamate (Hyperglutamate Theory) as compared to controls (Fatemi, 2008). The level of Glutamate (Glu), together with other potentially interesting molecules, including n-acetyl-aspartate (NAA), choline (Cho), myoinositol (mI) and creatine (Cr), was measured in the auditory cortex of subjects with ASD, pASD and HC. BAP traits in pASD were assessed by AQ and by Social Responsiveness Scale (SRS) (Constantino, 2002). While ASD subjects had increased levels of Glu compared with both pASD and HC, no differences were found between pASD and HC. Although not significantly different, the mean levels of the explored molecules in the pASD group were found to be intermediate between the HC and the ASD group. A significant positive correlation between left NAA and the SRS as well as between left Glu and the AQ was observed, but these correlations did not remain significant after multiple comparison correction. Both ASD and pASD did not exhibit sex differences in any of the MRS measures.

### Functional MRI (fMRI)

The first study that evaluated the BAP in pASD through fMRI technique was performed by Baron-Cohen et al. (2006). In this investigation, the authors used the visual search task “Adult Embedded Figures Test” (EFT) (Witkin et al., 1962), and the advanced emotion recognition task test “Reading the Mind in the Eyes” (or Eyes) (ET) (Baron-Cohen et al., 2001b) in order to see if the parents showed the same atypical brain function observed in the autistic children (Baron-Cohen et al., 1999; Ring et al., 1999). They also preliminarily explored the influence of sex on brain functioning during these two tasks in a small sample of six males and six females. Results indicated that pASD showed atypical brain activity compared with HC; moreover sex differences in neural underpinnings of both tests were found. As far as the EFT task is concerned, pASD showed less activity in the visual cortex while a reduced activity in the mid-temporal gyrus, and the inferior frontal gyrus was observed using the ET task.

As regards sex differences in the EFT, female controls displayed increased activity in middle occipital gyrus than male controls while both mothers and fathers showed even less activity in this area than sex-matched controls. In the ET, female controls exhibited more activity in the left medial temporal gyrus and left dorsolateral prefrontal cortex than male controls, while both mothers and fathers of children with ASD showed a brain activity similar to that of male controls. Mothers and fathers had comparable brain activation. One of the region identified as atypically activated in the ET task (B44) overlaps with a region previously identified as involved in “theory of mind” (Frith and Frith, 1999).

Greimel et al. (2010) explored in ASD boys and in their fathers (pASD) aspects related to the social domain of ASD, and in particular to the mechanism of empathy. Two aspects of empathy were evaluated related to (1) inferring how another person feels (other-task), and (2) responding appropriately to emotions of others (self-task). Comparison groups consist of age-matched typically developing boys (HC) and their fathers (pHC). Brain activation was analyzed in three predefined ROIs, the fusiform gyrus (FG), the inferior frontal gyrus (IFG) and the AMY and correlations with behavioral traits were evaluated. Empathic abilities were assessed by the Griffith Empathy Measure (GEM) in ASD and by the Balanced Emotional Empathy Scale (BEES) in pASD.

Despite a normal performance in reference to the number of correct/incorrect responses and even a faster response than pHC, pASD showed an abnormal brain activation. Specifically, both boys with ASD and their fathers obtained reduced anterior FG activation during the other-task, and boys with ASD additionally exhibited reduced FG activation during the self-task compared to HC. Interestingly, the activation within the FG occurred outside the well-known fusiform face area leading to exclude that differences of activation detected in this area were ascribable to a deficit in face processing. This hypothesis was corroborated also by the recording of the gaze during the fMRI task that showed an intact gaze pattern in scanning faces both in the adolescents with ASD and in their fathers. A diminished activation was also found in AMY in fathers of boys with ASD compared to control fathers when inferring others' emotions from weak cues, while in the ASD group this result was only obtained at an uncorrected threshold. The author hypothesized that fathers activated strategies to compensate for FG and AMY dysfunction. An involvement of the mirror neuron system (MNS) was also observed mainly in the ASD adolescents who showed a reduced activation of the IFG during the self-task. In both pASD and ASD groups a significant correlation between behavioral measures of empathy and brain activation was detected: specifically, in the ASD group the correlation was significant with activation of FG while in the pASD group with activation of the insula. However, no significant correlation was found between brain activity and AQ scores in pASD.

Together with social impairments, language dysfunction is another well-known hallmark of ASD. Extending the boundaries, language ability, specifically phonological processing ability, has been proposed to be one of six candidate BAP traits (Dawson et al., 2002).

Wilson et al. (2013) explored the neural correlates of phonological processing ability in a group of parents of children with ASD and in a group of age-matched controls. The task proposed consisted of prime-target word pairs differing in terms of their phonological relatedness including both word-word homophone and pseudoword-word pseudohomophone. Brain activation was also correlated with a behavioral measure of phonological processing ability obtained by the non-word repetition subtest of the Comprehensive Test of Phonological Processing (CTOPP) (Wagner et al., 1999).

Despite non-significant differences in terms of task performances and CTOPP scores and low AQ scores, pASD showed significantly higher hemodynamic responses than controls for pseudohomophone compared with homophone priming. Several cortical regions were involved in this abnormal activation, including the left anterior insular cortex (IC), the bilateral cerebellum and thalamus, left postcentral gyrus, precentral gyrus, and supplementary motor area (SMA), right superior temporal gyrus (STG) and supramarginal gyrus (SMG): interestingly, most of these regions had been previously implicated in language processing (Baddeley, 1992; Ackermann and Riecker, 2004; Hickok and Poeppel, 2007; Ghosh et al., 2008). Significant positive correlations were also observed between greater hemodynamic response and CTOPP in right STG, left IFG and IC in pASD and in several regions in controls (i.e. bilateral occipital gyrus, parietal lobule, postcentral gyrus, lingual gyrus, and IFG).

Moreover, parents of boys with ASD exhibited increased hemodynamic suppression in response to phonological priming compared with controls in several cortical regions including both the left lateralized STG and SMG. Both groups expressed a significant left lateralization in the ROI selected for the analysis.

The more recent fMRI study conducted in parents of individuals with ASD investigated neural substrates of face processing (Yucel et al., 2014). This is the only study which subset the parents on BAP traits. Specifically, in order to investigate the characteristics of a specific endophenotype linked to social behavior, the parents were classified in a group having “aloof personality” (BAP+) and a group having “non-aloof personality” (BAP−). The classification was based on the Broad Autism Phenotype Questionnaire (BAPQ) and the Modified Personality Assessment Schedule—Revised (MPAS-R) specifically designed to determine the presence or absence of “aloof personality.” Using two face activation paradigms, one based on face memory and the other based on emotional matching, the authors found that pASD had a higher activation of AMY and FG and a lower activation of right insula compared with HC, while no significant difference in activation was observed between BAP+ and BAP− in these regions. Conversely, BAP+ and BAP− parents significantly differ in terms of activation of the lateral occipital cortex (LOC). Indeed, BAP+ parents showed a bilateral hyper-activation in the LOC compared with both BAP− and HC.

### Neurophysiology (electroencephalography and magnetoencephalography)

The first electrophysiological study in pASD was performed by Dawson et al. (2005) who evaluated event-related brain potentials to face and non-face stimuli. Specifically, upright and inverted faces or chairs were presented to a group of pASD and HC and N170 amplitude and latency was measured at the inferior right and left posterior temporal regions. While HC showed the typical pattern of higher right than left N170 amplitude in response to faces (Bentin et al., 1996), pASD exhibited reduced right N170 amplitude resulting in bilaterally distributed brain activity to faces. In addition, HC had the expected faster N170 response to upright faces compared to upright chairs (Itier et al., 2006), while pASD showed no differences in latency in response to the two types of stimuli. Abnormalities in brain activity in pASD compared to controls were also associated to lower performances in behavioral tests (face recognition and object memory).

Subsequent studies explored brain activity in pASD in response to different stimuli using magnetoencephalography (MEG), focusing on high-frequency bands.

First, Rojas et al. (2008) investigated both evoked and induced components of the *transient gamma-band response (tGBR)*, elicited by auditory stimulation in subjects with ASD, in pASD and in a comparison group of healthy subjects. Source localization of the data was performed on MRI data acquired on the subjects enrolled in the study (Peterson et al., 2006). In addition to evoked and induced power, the authors also computed the *phase locking factor (PLF)* as a measure of phase consistency across trials.

Both pASD and the ASD groups showed bilaterally higher induced tGBR response compared with controls, while evoked tGBR was found bilaterally reduced in the same comparison. The PLF was also bilaterally reduced in both the pASD and the ASD group compared with HC. Moreover, both the pASD and the ASD group had a reduced anterior-posterior asymmetry of the magnetic sources compared with controls. In this study, no differences between pASD and ASD were found: such findings could be attributable to the low statistical power, but could also suggest that parents had the familial liability relevant to gamma-band disturbances.

Later, Rojas et al. (2011) extended the results of their previous work analyzing not only the tGBR component of gamma-band power, but also the *auditory steady-state response (ASSR*), in response to auditory stimulation. A group of pASD was compared with a control group of HC. In this study, authors also correlated MEG results with scores indicative of BAP−traits (AQ and SRS). The group of pASD exhibited reduced evoked power, total power (left hemisphere) and PLF (left hemisphere) of the ASSR component relative to the HC group. However, the authors were not able to replicate their previous findings relative to tGBR (Rojas et al., 2008), as they did not found any significant differences between pASD and HC.

Interestingly, an inverse correlation between ASSR PLF and the AQ communication subscale was found in pASD, confirming an association of gamma-band activity to perception of speech sounds and lexicality (Kaiser, 2004; Basirat et al., 2008). An inverse correlation was also observed in pASD between SRS scores and tGBR and ASSR gamma-band evoked power suggesting an indirect relationship between auditory gamma-band dysfunction and social traits of ASD.

In another investigation (McFadden et al., 2012), gamma-band response was analyzed in pASD and in HC in response to auditory language stimuli, rather than to simple auditory stimuli. In this contest, beta band activity was also examined since it has been suggested to be involved in language processing (Shahin et al., 2009). While in the previous two investigations (Rojas et al., 2008, 2011) pASD showed decreased evoked gamma-band response compared with HC, in this study pASD exhibited increased evoked power. In addition, there was an increase in pASD of total gamma power compared with controls. Source localization analysis showed that this increase was mainly localized in the SMG, in the lateral occipital cortex (LOC), and in the FG.

Beta evoked activity was also found increased in pASD compared with controls mainly in SMG, but also in LOC and FFG possibly reflecting differences in cognitive function during language processing. While in both groups the task generally elicited left lateralized responses, pASD showed greater left lateralization than controls, confirming also in this case an atypical lateralization of the brain in pASD. Significant but different correlations were found between gamma or beta band activity and language measures.

Gamma and beta band responses were also assessed in pASD compared with HC during a picture-naming task (Buard et al., 2013). Subjects were instructed to sub-vocalize (to reduce motion artifacts) the name of the object depicted in the image they were shown. Due to their involvement in language function and in visual processing, FG, STG, IFG and occipital lobe (OCC) were considered as the regions of interest. As in the three previous studies (Rojas et al., 2008, 2011; McFadden et al., 2012), evoked and induced power together with PLF were computed. In addition, Granger causality function, as a measure of effective connectivity among the activated regions, was measured.

Interestingly, the ASD group and the pASD showed different patterns of activation both in gamma and beta bands. While the ASD group exhibited reduced evoked high-gamma activity in the right STG, increased evoked high-beta/low-gamma in the left IFG and reduced PLF beta in the OCC, the pASD group showed increased evoked high-gamma in the left STG and evoked high-beta/low-gamma in the left FG.

Functional connectivity abnormalities were only observed in the ASD group compared with the control group: specifically, over-connectivity was found in the left hemisphere between IFG and FG and between STG and OCC in both gamma and beta band. This altered functional connectivity from anterior to posterior language and visual areas may partially explain the impaired activation of these regions in the ASD group, ascribable to alterations in long-range neural synchronization.

## Discussion

The main leading hypothesis tested in this review is that pASD present with a number of neuroanatomical and neurofunctional characteristics observed in individuals with ASD, but to a lesser extent. This hypothesis is supported by previous studies demonstrating intermediate levels of biochemical, immunological, morphological and neuropsychological endophenotypes/biomarkers in pASD (Ruggeri et al., 2014).

In some cases, the results of the studies, using different methodological approaches, have supported the primary hypothesis, while in other cases different results have emerged.

### Are pASD different from HC?

All of the 13 reviewed studies compared the pASD with a sample of HC.

- No differences in total brain volumes between pASD and controls were found in any of the three sMRI studies (Rojas et al., 2004; Palmen et al., 2005a; Peterson et al., 2006). This finding is not surprising when considering that even within the ASD population many/most adults, unlike children, do not differ from controls in overall brain volume. Indeed, there is increasing evidence that brain growth trajectory is abnormal in subjects with ASD and that they have differences in the timing of both initiation and cessation of overall brain growth, resulting in larger brain volumes during childhood followed by later normalization (Courchesne et al., 2001, 2003; Dawson et al., 2007).
- More inconsistent findings were reported for the single brain structures. While Palmen et al. (2005a) found no differences between pASD and HC groups in any of the volumes considered, including cortical lobes, cerebral GM and WM, cerebellum, and ventricles, increased volumes were found in the left hippocampus (Rojas et al., 2004) or in a number of GM regions (Peterson et al., 2006). The different approach in analyzing brain regions (global brain structures—Palmen et al., 2005a—vs. focal structures—Rojas et al., 2004—vs. whole brain approach—Peterson et al., 2006) prevents a comparison among studies. Overall, the inconsistency of these results reflects that of the studies on subjects with ASD (Ameis and Catani, 2015). Methodological differences between investigations and the potential for heterogeneity of underlying brain alterations in ASD likely contribute to the inconsistency of these results.
- Functional studies showed some atypicalities in face processing, empathy and language/auditory processing in pASD compared with HC.

#### Face

The study by Dawson et al. (2005) support the social motivation impairment showing an abnormal N170 response to faces both in its latency and amplitude with a pattern resembling that observed for subject with ASD (Apicella et al., 2013). Yucel et al. (2014) observed an increased activation in pASD compared with controls during an emotion recognition task in regions that are specialized for face processing, i.e., the fusiform gyrus and the amygdala.

#### Empathy

An opposite pattern was found by Greimel et al. (2010) who explored empathy during the presentation of emotional stimuli in pASD and found a decreased activation in the same regions. It is possible that the two different types of task lead to different brain activations. Moreover, the different results could be explained by the fact that the sample of Greimel study is composed of males only who are generally less empathic than females (Klein and Hodges, 2001), and therefore process emotion to a lesser extent than females. In addition it is worth noting that Yucel et al. (2014) found a decreased activation of the insula, which is known to be linked to empathic abilities (Carr et al., 2003) as also suggested by the positive correlation found in Greimel et al. (2010) between insula activation and the BEES. Emotion recognition impairments in pASD also emerged from the study of Baron-Cohen et al. (2006) who observed a decreased activation in the left IFG of pASD compared with controls during an emotion recognition test.

#### Language

Wilson et al. (2013) showed that pASD compared with HC exhibit a greater hemodynamic response to pseudohomophones respect to homophones and an enhanced hemodynamic suppression in response to phonological priming. Interestingly, Peterson et al. (2006) observed both cerebellar enhancements and reductions, although in different cerebellar regions than those differently activated in the phonological task, and larger left STG and SMG GM volumes in pASD relative to HC. These regions are known to be involved in language and phonological processing (Turkeltaub and Coslett, 2010) and to be functionally impaired in ASD (Mostofsky et al., 2009).

Abnormalities associated to language processing have been shown also by MEG studies, mainly associated to gamma-band response. In particular, gamma-band deficit, which has been suggested as a biomarker of ASD (Jamal et al., 2013; Rojas and Wilson, 2014), exists also in pASD, and abnormalities seem to extend also to the beta band. In ASD individuals, dysfunctional gamma-band response has been associated with GABAergic inhibitory deficits (Hussman, 2001; Fatemi et al., 2009). Conversely, multiple evidences suggest an increased neuronal excitability in ASD, involving a higher than normal serum glutamate (Shinohe et al., 2006), and increased metabotropic glutamate receptor expression (Fatemi et al., 2011). Overall, these evidences have been summarized in the excitation/inhibition imbalance (EI) theory of ASD (Rubenstein and Merzenich, 2003).

Rojas et al. (2008, 2011) explored gamma band response to auditory stimulation in pASD. Induced response has been found increased in pASD (Rojas et al., 2008), while evoked response and PLF were decreased (Rojas et al., 2011) compared with HC in response to simple auditory stimulus. However, more complex stimuli activate a different pattern as observed in subsequent investigations (McFadden et al., 2012; Buard et al., 2013). In both these studies, pASD showed an increased evoked gamma band response compared with HC, which extended also to beta band in Buard et al. (2013). The different findings among these studies might be explained by the different level of complexity of the tasks: specifically, subjects were requested to be engaged in higher order cognitive processes including language and sustained attention (McFadden et al., 2012; Buard et al., 2013), or only passive listening to a simple auditory stimulus was required (Rojas et al., 2008, 2011).

The studies exploring auditory/language processing suggest that when pASD are involved in higher cognitive function they activate a higher brain response compared to that of controls, possibly as a compensatory mechanism in absence of behavioral impairment. In the study by Wilson et al. (2013) the greater hemodynamic responses in the parent group might reflect the heavier demands requested by the pseudohomophone primes on phonological recoding and working memory skills compared with homophone primes, and it can be interpreted as an index of more effortful processing during this task. Analogously, in the studies by McFadden et al. (2012) and Buard et al. (2013) the increase in gamma and/or beta could reflect a greater cognitive effort in phonology and receptive language tasks, which determine an abnormal synchronous activation of language networks (Jerbi et al., 2009).

It is worth noting that functional abnormalities at a neural level in pASD are not always associated to behavioral impairments. For example Greimel et al. (2010) found a non-compromised empathic ability in an emotion recognition task while Wilson et al. (2013) found no difference in terms of phonological processing (CTOPP scores) between pASD and HC. Conversely in the study by Dawson et al. (2005) the authors found that neurofunctional abnormalities and neuropsychological performances in pASD were associated, suggesting that pASD are more compromised at a neural level than at a behavioral level.

### How are parents of individuals with ASD compared to other individuals with ASD?

Only five of the 13 studies addressed the question of the overlap between pASD and other individuals with ASD (Rojas et al., 2004, 2008; Greimel et al., 2010; Brown et al., 2013; Buard et al., 2013). All reported similarities in some aspects of brain structure and function consistent with the hypothesis of a continuum of some ASD features expressed in pASD, with milder but qualitatively similar brain alterations to those detected in ASD.

In particular, structural (Rojas et al., 2004) and spectroscopy (Brown et al., 2013) studies revealed a brain endophenotype in pASD intermediate between ASD patients and HC. Specifically, Rojas et al. (2004) found that hippocampus enlargement interested also pASD, but to a lesser extent than ASD individuals. Vice-versa, the amygdala was smaller in ASD patients compared to pASD. Brown et al. (2013) showed that the mean levels of the explored molecules in the parent group were intermediate between ASD individuals and HC. However, these findings were not statistically significant possibly due to the small sample size and/or to the low scores at the AQ and the SRS of the pASD subjects.

The fMRI study exploring the neural correlates of empathy (Greimel et al., 2010) reported a reduced activation in the right anterior fusiform gyrus in both adolescents with ASD and pASD compared to age and IQ matched controls. Finally, using EEG a reduced early auditory gamma-band response shared by both adults with ASD and pASD in comparison to HC was detected (Rojas et al., 2008).

Several other patterns of brain activity were not shared by pASD and ASD patients, potentially suggesting a lesser role of these aspects as endophenotypes of the disorder. For example, in the same fMRI study exploring empathy (Greimel et al., 2010), reduced amygdala activity was found in pASD but not in ASD. Also, using MEG during a picture naming task, Buard et al. (2013) found that gamma-band activity showed opposite profiles in pASD and in ASD subjects relative to controls, being increased in the former and reduced in the latter.

All in all, the limited number of studies addressing this question does not allow for definitive conclusions although it seems to be conceivable that some aspects of brain structure and function are shared by pASD and ASD patients, supporting their possible role as endophenotype of the disorder.

### How are parents of individuals with ASD compared to their probands?

The study by Greimel et al. (2010) was the only one that enrolled both the probands and their fathers to explore the transmission of neural substrates. The results confirmed the primary hypothesis of a neurofunctional pattern in pASD intermediate between HC and ASD. In particular, pASD showed an abnormal neural activation during the other-task similar to their probands, expressed by a reduced hemodynamic response in FG – a tempo-occipital brain region primarily involved in face processing-. Conversely, unlike their probands, pASD showed a normal response during the self-task. It is of interest that a reduced activation of regions previously associated to the MNS, namely IFG, was found in ASD probands but not in their fathers. These results support the hypothesis that FG dysfunction in ASD is genetically influenced (Polk et al., 2007).

### Are the neurostructural and neurofunctional alterations reported in parents of individuals with ASD specifically related to BAP features?

Six of the reviewed studies (Palmen et al., 2005a; Greimel et al., 2010; Rojas et al., 2011; Brown et al., 2013; Wilson et al., 2013; Yucel et al., 2014) assessed the BAP characteristics of the pASD applying instruments such as the AQ, the BAPQ or the SRS. Significant correlations between scores at the questionnaires and the brain structural and functional indexes were found in almost all these studies. In particular, Palmen et al. (2005a) found a significant positive correlation between AQ scores and intracranial and ventricular volume in pASD, while Brown et al. (2013) found a significant, uncorrected, positive correlation between left NAA and the SRS and left Glu and the AQ. Both studies did not report significant differences in pASD compared to HC (Palmen et al., 2005a; Brown et al., 2013); however, the fact that pASD scored very low at questionnaires assessing BAP could represent a possible bias leading to negative findings. Interestingly, in both studies a positive correlation between neurostructural results and BAP features was found, suggesting that enlarged brain volume or increased Glu level respectively could still be associated to the autistic phenotype.

Notably, Wilson et al. (2013) found significant differences in brain activation during a language task in the pASD sample, despite low scores at the AQ. This result could suggest that deficits in neural substrates of language processing could be associated with autistic traits, confirming it as one of the core impairment of ASD.

This is confirmed by the investigation of Rojas et al. (2011) in which a negative correlation between ASSR PLF and AQ communication subscale as well as between SRS scores and tGBR/ASSR evoked power was observed. ASSR PLF and evoked power were found decreased in pASD compared to HC: therefore, it can be argued that deficits in auditory gamma band are correlated to problems in communications (AQ) and socials skills (SRS). Despite the authors did not find significant differences regarding tGBR, a significant correlation between SRS scores and this feature was observed in pASD suggesting a possible association with the autistic phenotype, as proposed in their previous study (Rojas et al., 2008).

Greimel et al. (2010) did not find any significant correlation between brain activation and AQ in pASD, while a brain-behavior relationship was detected with empathic scores (BEES).

The paper by Yucel et al. (2014) was the only one subgrouping the parent sample according to BAP traits. Interestingly, the authors observed that while an atypical activation of face processing regions was common to both groups of parents, BAP+, but not BAP− parents showed an hyper-activation of lateral occipital cortex. The hyper-activation of LOC in BAP+ could reflect an aberrant “compensatory” activation of these regions in BAP+ parents. These data suggest that while neural circuitry abnormalities in the regions specific for face processing are necessary for the occurrence of the BAP, they are not sufficient to result in autism-related social behavior.

Overall, these findings suggest a possible link between the subclinical dimension of BAP and neurobiological expression of brain function and structure.

### Does gender influence neurostructural and neurofunctional results in parents of individuals with ASD?

Previous studies have reported sex differences in brain in healthy populations and these processes have shown to differ in people with ASD. In particular, sexual dimorphism in brain regions that are crucial to language and social abilities has been proposed (Lai et al., 2013; Retico et al., 2016).

Understanding cerebral gender differences is important, among other reasons, to explain the increased vulnerability of males to ASD. Few studies have explored gender differences in pASD in order to investigate the heritability of sex differences in brain structures and function.

From a structural point of view, it was observed that males, both pASD and HC had increased total, hippocampal and amygdala volumes (Rojas et al., 2004), as well as GM (Peterson et al., 2006) compared to pASD and HC females, but this difference did not contribute to between-groups differences. Brown et al. (2013) investigated gender differences in MRS measures both in ASD and pASD and did not find any differences as well. Conversely, Baron-Cohen et al. (2006) found significant differences in brain function related to gender. In particular, their results support the hypothesis of the “Extreme Male Brain Theory” of ASD, according to which “the male brain is programmed to systemize and the female brain to empathize” (Benenson, 2003). Indeed, both mothers and fathers showed an activation even lower than that of male controls in regions were female controls had a higher activity. The results of this study may suggest a genetic component of the hyper-masculinization of the brain.

### Do parents of individuals with ASD express an atypical lateralization of the brain?

In typical development, lateralization of brain function underlies specialized cognitive and behavioral processes (Mesulam, 1990). In particular, several pieces of evidence exist about a left lateralization in language regions (Knecht et al., 2000), and right lateralization in attentional regions (Corbetta and Shulman, 2011) in the majority of individuals with typical development. Atypical lateralization in brain structure and function has been associated with ASD (Conti et al., 2016); more specifically, reduced left lateralization or reversed lateralization of brain structure and function in core language regions and in the WM tracts that connect them has been shown in ASD using different techniques (Kleinhans et al., 2008; Lange et al., 2010; Seery et al., 2013).

Whether the pattern of lateralization related to language processing observed in subjects with ASD is the same in their parents is not clear from the reviewed studies.

In Wilson et al. (2013), hemispheric lateralization analysis did not indicate greater right hemispheric language dominance in the pASD: in fact, both pASD and HC showed left lateralization across the selected ROIs. McFadden et al. (2012) found that pASD showed even an increased left lateralization than controls. Rojas et al. (2011) showed that differences in ASSR response in pASD was restricted to the left hemisphere, however across groups the tGBR and ASSR evoked power was increased in the right hemisphere. Rojas et al. (2008) highlighted a peculiar pattern of asymmetric activation in control subjects in which the activation of the right hemisphere was anterior to that of the left hemisphere. This pattern was not observed in ASD and was mild in pASD.

Yucel et al. (2014), observed in the face processing task a significant effect of hemisphere. Specifically, FG showed greater activation in right than left hemisphere in BAP+ compared with BAP− and HC, and right amygdala was more active than left in BAP+ compared with BAP−. Since right lateralization was observed specifically in BAP+ pASD, a compensatory mechanism of activation in these regions could be hypothesized for this subgroup of parents. Additional support for this interpretation can be found in the investigation of Rojas et al. (2004) on the basis of which pASD has reduced right amygdala volume compared with HC: it may be possible that to compensate the reduction of volume an abnormal high activation is required.

## Conclusions and future directions

Although, results are often unclear and contradictory, some general considerations can be done:

- pASD differ from HC both at a structural and functional level and these neural abnormalities are not always associated with behavioral impairments;
- The neural pattern in pASD seems to be intermediate between HC and ASD probands;
- More atypicalities in neural patterns of pASD seem to be associated with higher autistic traits;
- The pattern of neural correlates in pASD resembles that of adult individuals with ASD or it is specific to pASD, possibly due to a compensatory mechanism;
- The gender might influence the results.

In conclusion, our review reports findings that are often non-replicated, preventing a univocal interpretation of the results.

**Figure 1:**
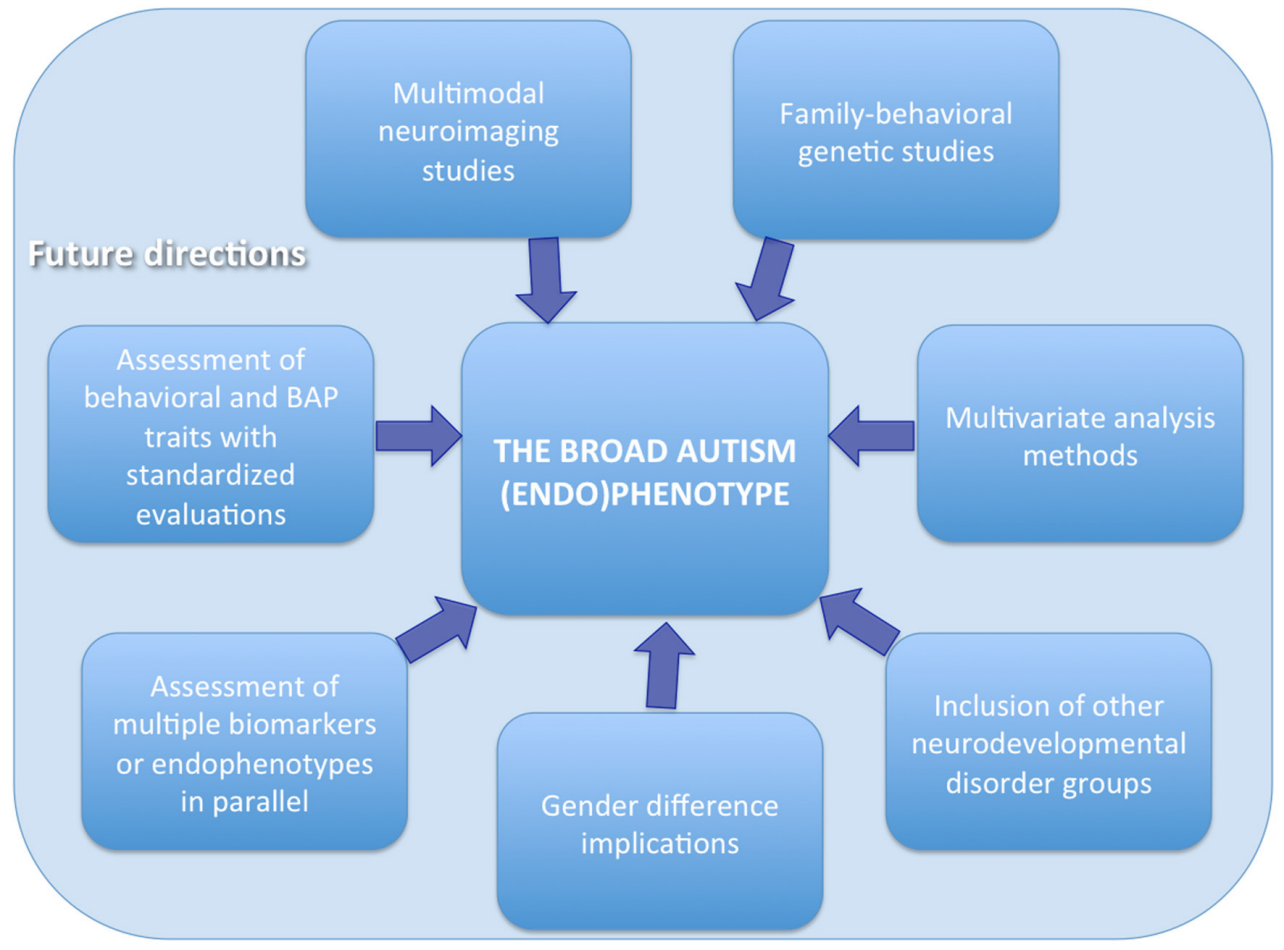
**Recommendations for future studies assessing the broader autism (endo)phenotype**.

In order to elucidate the brain structural and functional underpinnings in pASD and their potential role as endophenotype, several aspects should be considered when planning future studies (Figure 1).

First, neuroimaging studies should be ideally include a group of pASD and HC with their ASD and non-ASD probands, respectively. Second, a family-behavioral genetic design should be adopted in order to analyze the behavioral features as well as the genetics not only of the probands, but also of the parents and the siblings, and to link these data to underlying brain structure and function. Third, behavioral assessment as well as BAP traits evaluation should be performed using standardized questionnaires and tests in order to subgroups the probands and their relatives according to the obtained scores and to investigate a possible correlation between brain abnormalities and BAP traits and/or behavioral impairments. Fourth, multimodal imaging techniques could also be adopted to better elucidate brain correlates of BAP. For example, the integration of neuroimaging data with neurophysiological signals (EEG and MEG) offers advantages of both high spatial and temporal resolution (Ingalhalikar et al., 2012; Berman et al., 2016). The application of these methods also in pASD probands could provide new insights into the endophenotype of ASD. Fifth, since gender can influence neural substrates, this factor needs to be carefully taken into account when grouping samples and interpreting the results. Indeed, brain endophenotypes could be related to differences in the developmental, psychiatric, and medical endophenotypes between males and females with ASD. These research findings may in turn help the clinical assessment and treatment of ASD and the search for possible etiologies (Rubenstein et al., 2015). Sixth, studies on the BAP could also benefit of the assessment of multiple endophenotypes/biomarkers in parallel by collecting, in addition to neuroimaging data, immunological, biochemical, or neuropsychological data and evaluating the cross talk among the different modalities (Ruggeri et al., 2014). Seventh, the inclusion of samples with other neurodevelopmental disorders rather than ASD can help to disentangle the specific from the non-specific endophenotypes associated to each condition. Indeed brain alterations have been found in relatives of probands with Attention Deficit/Hyperactivity Disorder—ADHD—(Casey et al., 2007; Hale et al., 2010; Poissant et al., 2014; Rapin et al., 2014), language impairments (Plante, 1991; Ors et al., 2002) and learning or intellectual disabilities (Mannerkoski et al., 2009). In particular, previous literature suggests that there are cognitive and brain endophenotypes common to ASD and ADHD and that studying the similarities and differences between these two disorders might be a powerful research approach to increase our understanding of their pathophysiology (Rommelse et al., 2011). Finally the use of multivariate approaches, based for example on machine learning (Retico et al., 2014; Segovia et al., 2014), can provide more insightful results than the traditional statistical analysis methods.

In conclusion, these types of implementations may help to better elucidate the hereditary mechanisms involved in the various clinical dimension of ASD.

## Author contributions

LB performed the literature-search, analyzed the data and drafted the paper, SC, EC made substantial contribution in the literature analysis and in writing the paper, CG, CC collaborated during the literature analysis, LD, GC participated in the design of the paper and supervised the wiring, FM, AG made substantial contributions to the conception of the review, and revisited the manuscript critically. All the authors read and approved the final version of the manuscript.

## Funding

The research leading to this work has received funding from the University of Pisa, Bando PRA 2015, Project “Broader autism phenotype: brain connectivity biomarkers from the adult to the child.” This work was also supported by grant from the IRCCS Stella Maris Foundation (Ricerca Corrente, and the “5 × 1000” voluntary contributions, Italian Ministry of Health to SC, GC, FM, AG). SC was supported by the Ministry of Health, Italy and by Tuscany Region with the grant “GR-2010-2317873,” and by Bando FAS Salute Sviluppo Toscana -ARIANNA Project.

### Conflict of interest statement

The authors declare that the research was conducted in the absence of any commercial or financial relationships that could be construed as a potential conflict of interest.
